# Transcriptome analyses of differential gene expression in the bursa of Fabricius between Silky Fowl and White Leghorn

**DOI:** 10.1038/srep45959

**Published:** 2017-04-13

**Authors:** Deping Han, Yuanyuan Zhang, Jianfei Chen, Guoying Hua, Junying Li, Xuegong Deng, Xuemei Deng

**Affiliations:** 1 National Engineering Laboratory for Animal Breeding and Key Laboratory of Animal Genetics, Breeding, and Reproduction of the Ministry of Agriculture, China Agricultural University, Beijing 100193, China; 2 College of Veterinary Medicine, China Agricultural University, Beijing 100193, China; 3 College of Science, Northeastern University, Shenyang 110004, China

## Abstract

Hyperpigmentation in Silky Fowl (SF) results in aberrant immune cell development. However, how melanocytes regulate B-cell proliferation in the bursa of Fabricius (BF) is unclear. To resolve this conundrum, we collected BFs from three-week-old SF and White Leghorn (WL) female chickens for RNA sequencing. The BF development was relatively weaker in SF than in WL. The transcriptome analyses identified 4848 differentially expressed genes, 326 long noncoding RNAs (lncRNAs), and 67 microRNAs in the BF of SF. The genes associated with melanogenesis was significantly higher, but that of the genes associated with the cytokine-cytokine receptor interactions and JAK-STAT signalling pathway was significantly lower in SF than in WL. Crucial biological processes, such as the receptor activity, cell communication, and cellular responses to stimuli, were clustered in SF. The predicted target lncRNAs genes were mainly associated with cell proliferation pathways such as JAK-STAT, WNT, MAPK, and Notch signalling pathways. Except for the above pathways, the target microRNA genes were related to the metabolism, melanogenesis, autophagy, and NOD-like and Toll-like receptor signalling pathways. The lncRNAs and microRNAs were predicted to regulate the *JAK2, STAT3,* and *IL-15* genes. Thus, B-cell development in the BF of SF might be regulated and affected by noncoding RNAs.

Silky Fowl (SF) is one of the most important animal models used to investigate melanocyte migration and genes related to hyperpigmentation^1^,^2^,^3^,^4^,^5^. Previous studies have shown that the ectopic pattern of pigmentation in SF results from an abnormal migration of melanoblasts. Different from the melanoblast migration that only takes the dorsolateral pathway between the ectoderm and somites in White Leghorn (WL), the melanoblasts in SF can migrate ventrally between the neural tube and somites, in addition to the dorsolateral migration^1^. Many studies have compared SF and WL to investigate the genes associated with the hyperpigmentation^3^,^4^,^5^. However, a few studies have focused on the function of melanocytes in different inner organs in SF, leading to a better understanding of the pathogenesis of human diseases such as melanoma and hypopigmentation. Some studies have reported that the melanocytes in humans and other animals participate in important biological processes. For instance, the perivascular-resident macrophage-like melanocytes in the inner ear maintain the integrity of the interstitial fluid-blood barrier by regulating the expression of several tight junction-associated proteins^6^. The epidermal melanocytes protect the skin from ultraviolet radiation by shielding DNA from damage^7^,^8^. In zebrafish, the inflammation caused by trauma attracts the melanocytes and melanoblasts to the site of injury after the initial recruitment of cells associated with the innate immune system, suggesting that the cytokines produced by immune cells induce melanocyte functions that mediate wound repair^9^. Furthermore, it has been reported that the aberrant melanocyte development and/or dysfunction is associated with multiple diseases, including albinism, Waardenburg syndrome, and piebaldism^10^.

High-throughput sequencing technologies are frequently used to explore crucial genes and noncoding RNAs, which play important roles in morphogenesis. Liu *et al*.^11^ reported that the differentially expressed genes associated with bursal development were found at different developmental stages using RNA sequencing (RNA-seq) analyses^11^. Wang *et al*.^12^ showed that the long noncoding RNA (lncRNA), lnc-DC, inhibits STAT-3 dephosphorylation in the JAK-STAT signalling pathway, thus controlling human dendritic cell differentiation^12^. Furthermore, a recent study found that the exosomes carrying keratinocyte-secreted microRNAs could be phagocytized by the melanocytes that modulate melanin synthesis, suggesting that the intercellular regulation of functions can be accomplished by noncoding RNAs^13^. The results of our previous study revealed that the hyperpigmentation in SF resulted in aberrant B-cell development in the bursa of Fabricius (BF)^14^. Therefore, the RNA-seq analyses were used to examine the differential gene expression in the BF of SF and WL in order to enhance our understanding of the possible roles of melanocytes in the modulation of B-cell development in the BF microenvironment.

## Results

### Weaker BF development in SF

**Figure 1:**
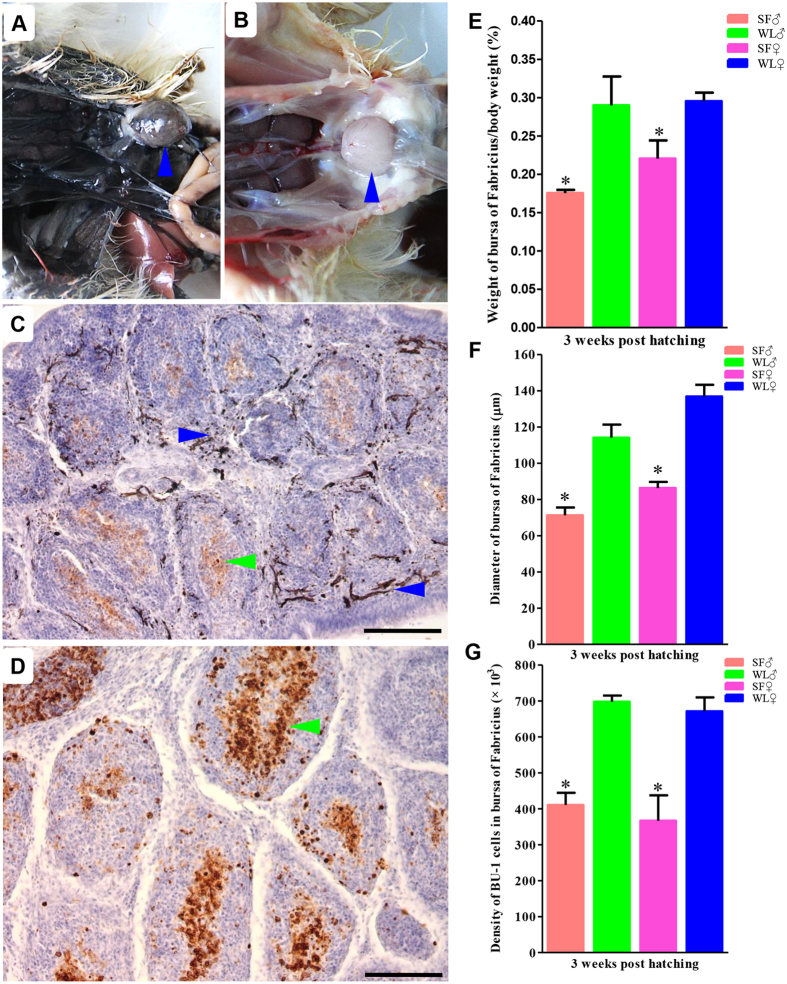
Weaker BF development in SF. Obvious hyperpigmentation was observed in the BF of SFs (**A**) compared to the WL chickens (**B**); fewer Bu-1^+^ cells (green arrow) and melanocyte (blue arrow) were detected in the BF of female SFs (**C**) than that of the WL chickens (**D**). The BF index was relatively lower in SF than in WL (**E**); Significant differences in the diameters of bursal nodes were detected between SF and WL (**F**); calculated statistical significance of Bu-1^+^ cells (**G**). Bar = 100 μm. *P < 0.05.

After dissection, the obvious hyperpigmentation was observed in the BF of SF (Fig. 1A–B). The BF index of SF was significantly lower than that of the WL chickens (P < 0.05, Fig. 1E). The bursal node diameters differed significantly between the SF and WL chickens (P < 0.05, Fig. 1F). In contrast to the WL chickens, significantly fewer Bu-1^+^ cells were observed in the BF of female SF (P < 0.05, Fig. 1C–D and G).

### Identification of differentially expressed genes in BF

**Figure 2:**
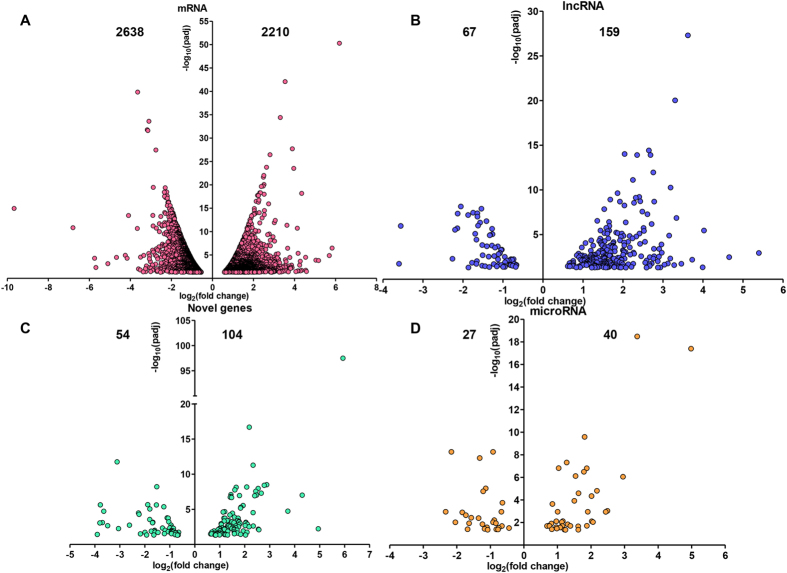
Number of differentially expressed genes, lncRNAs, and microRNAs in BF. Compared to the WL chickens, the expression of 2210 coding genes was higher and that of 2638 genes was lower (**A**); that of 159 lncRNAs was higher and that of 67 was lower (**B**); that of 104 novel genes was higher and that of 54 was lower (**C**); and that of 40 microRNAs was higher and that of 27 was lower (**D**) in SF.

After the analysis of the gene expression profiles of BF, approximately 50 million raw reads were generated for each strain, and 45–51 million clean reads were obtained for further analyses. Over 75% of the clean reads mapped to the reference genome, and the correlation coefficients of gene expressions for the three biological replicates ranged from 0.975 to 0.987 (Fig. S1). Following transcriptomic analyses, under the P_adjust_ < 0.05 conditions, 5174 (4848 mRNAs and 326 lncRNAs) differentially expressed genes (DEGs) were identified in the BF based on the comparisons between SF and WL chickens (Table S1). Moreover, significant differences were detected in 158 novel genes between SF and WL chickens. Comparisons between SF and WL chickens revealed 67 differentially expressed microRNAs in the BF. An increase in the expression of 2210 mRNAs and a decrease in the expression of 2638 mRNAs was observed in the BF of SF (Fig. 2A). The differential expression of lncRNAs (including 159 with relatively higher and 67 with relatively lower expression) and novel genes (including 104 with relatively higher and 54 with relatively lower expression) was detected (Fig. 2B–C). Of all differentially expressed BF microRNAs, 40 showed an increase and 27 showed a decrease in expression (Fig. 2D).

**Figure 3:**
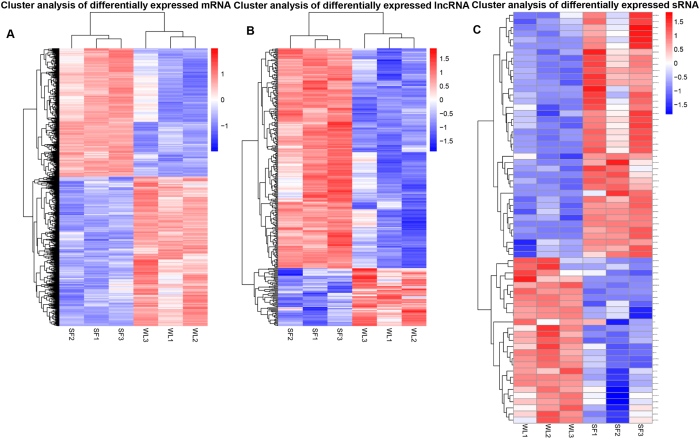
Clustering analysis of DEGs, lncRNAs, and microRNAs. The hierarchical clustering of DEGs (**A**) and lncRNAs (**B**) based on the values of log_10_ (FPKM + 1). Hierarchical clustering of differentially expressed microRNAs (**C**) based on the data from log_10_ (TPM + 1). Red and blue indicate the higher and lower expression levels, respectively.

Using the hierarchical cluster analysis, distinct expression patterns of mRNAs and lncRNAs were detected in the BF of SF and WL (Fig. 3). Additionally, significant differences were observed between the microRNA expression patterns of SF and WL (Fig. 3C).

### Functional analysis of DEGs and noncoding RNA target genes

The differentially expressed mRNAs were assigned to 302 functional subcategories, mainly associated with the biological processes and molecular functions (Fig. S2A). The former included the signal-organism processes, signal-organism cellular processes, biological regulation, cell communication, signal transduction, regulation of biological processes, signalling, regulation of cellular processes, response to stimulus, and biological adhesion. The latter included the receptor activity, sequence-specific DNA binding, molecular transducer activity, nucleic acid binding transcription factor activity, sequence-specific DNA binding transcription factor activity, signalling receptor activity, transmembrane signalling receptor activity, chemokine activity, and chemokine receptor activity. The cellular membrane components, intrinsic to membrane, integral to membrane, extracellular matrix, and membrane parts were also clustered.

The differentially expressed mRNAs associated with the metabolic pathways within the KEGG pathway database were analysed, and 26 pathways were found to be overrepresented in the BF of SF (Fig. S2B). For example, the cytokine-cytokine receptor interaction, ECM-receptor interaction, focal adhesion, and cellular adhesion molecule pathways were significantly overrepresented (P_adjust_ < 0.05). In addition, the lysosome, phagosome, JAK-STAT signalling, melanogenesis, DNA replication, arachidonic metabolism, cytochrome P450-mediated xenobiotic metabolism, and actin cytoskeleton regulation pathways were overrepresented in the BF of SF. The genes associated with cell proliferation pathways having P_adjust_ < 0.05 included *IL21/IL21R, GH/GHR, IGF2/IGF2R, TGFB/TGFBR, EGFR, IGF1R, JAK2, STAT1/3/4/5B, AKT1, RAF1, MAPK, CAMK2A, FASL/FAS, BCL-2, TNFSF NFIB, IKBIP, NFKBIA/2, TCF7, JUN*, and *WNT*. In addition, the differentially expressed genes related to innate immune response (*TLR1B/3/4/5/15, NOD1*, and *IRF*) and melanogenesis (*EDN3/EDNRB, TYR/TYRP1, KIT/KITLG, MITF*, and *MC1R*) were detected in the BF of SF.

Following the pathway analysis, the gene ontology (GO) terms (P_adjust_ < 0.05) of the target lncRNA genes were found to be mainly related to the biological processes, including cell communication, regulation of cellular processes, and single-organism cellular processes. The cellular components were associated with the nuclei, cytoskeletons, and organelles, whereas the molecular functions mostly referred to the protein binding, transcription factor, and receptor activities. The result of the KEGG analysis revealed that the enriched pathways were involved in DNA replication, adherens junction, Notch signalling, JAK-STAT signalling, Wnt signalling, and lysosome pathways (Fig. S2C, D). The GO terms of the target microRNA genes were also mainly associated with the cell communication, signal transduction, protein binding, and receptor activities. The significantly enriched KEGG pathways were involved in the Notch signalling, NOD-like receptor signalling, endocytosis, and tyrosine metabolism pathways.

### Quantitative PCR validation of differentially expressed genes

To validate the RNA-seq results, five mRNAs, five microRNAs, and five lncRNAs from the BF (lncBF) were selected and quantified using quantitative PCR (qPCR). As shown in Fig. S3, the relatively high expressions of these genes were further confirmed by qPCR. A high correlation, with the Pearson’s correlation coefficient of 0.834 (P < 0.0001), was observed between the qPCR and RNAseq results.

### Close relationships between mRNA, lncRNA, and microRNA

**Figure 4:**
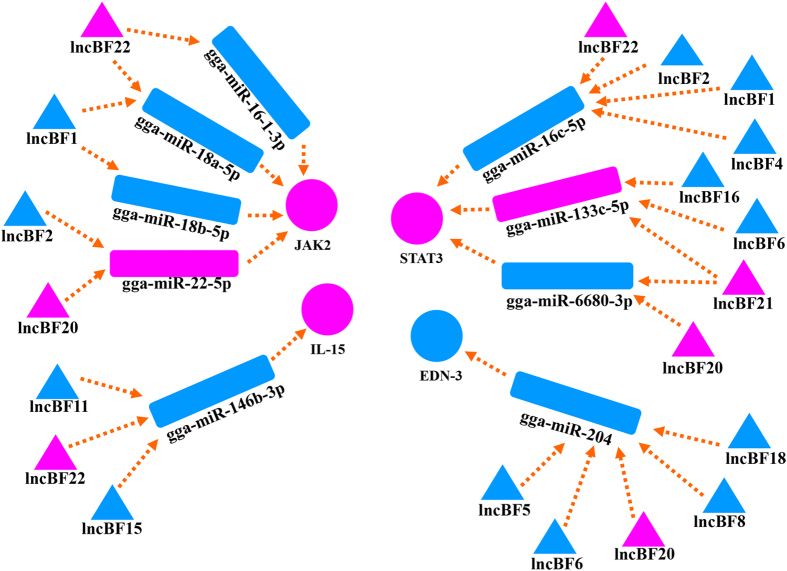
Predicted interactions between lncRNAs and *JAK2, STAT3, IL-15*, and *EDN-3*. Blue and purple indicate the higher and lower expression levels, respectively. lncBF refers to the lncRNAs expressed in the BF.

After the analysis of lncRNAs, which are thought to be the potential pre-microRNAs, the interaction network of the microRNAs, mRNAs, and lncRNAs was predicted using the miRanda software. Compared with the WL chickens, 258 differentially expressed microRNAs were upregulated and 68 targeted lncRNAs were downregulated in SF, and of the targeted mRNAs, 2181 were upregulated and 2611 were downregulated. Because of the competitive combination with microRNAs, the interaction network of the lncRNAs-microRNAs-mRNAs was significantly predicted, and it showed overexpression of microRNAs with downregulated lncRNAs and mRNAs, or decreased expression of microRNAs with upregulated lncRNAs and mRNAs. Using microRNAs as bridges (Fig. 4), the lncRNAs that interacted with the poorly expressed *JAK2, STAT3*, and *IL-15* genes were predicted. The highly expressed lncRNAs included *lncBF1, lncBF2, lncBF4, lncBF6, lncBF10, lncBF11, lncBF15*, and *lncBF16*, and the poorly expressed lncRNAs included *lncBF20, lncBF21*, and *lncBF22. EDN-3*, which is closely associated with melanocyte migration, was predicted to interact with the four highly expressed lncBFs and one poorly expressed lncBF using the stem-loop sequence gga-miR-204.

## Discussion

Hyperpigmentation in the inner organs of SF provides a good model for investigating the molecular mechanisms associated with melanocyte migration and function in different tissue microenvironments. Previous studies have shown that melanocytes in the skin and inner organs of SF are mainly distributed around the blood vessels and mast cells^15^,^16^. Our previous work also indicated the same characteristic distribution of melanocytes in different SF organs, and we found that the hyperpigmentation plays an important role in immune system development^14^,^17^. A previous report has demonstrated that the three-week age is very important for the development of B-cells and provides an opportunity for some pathogenic infection^18^. We also found that the development of BF was significantly weaker in SF than in WL^14^. In the present study, numerous melanocytes were observed in the lamina propria and bursal nodes of BF in SF. These results suggested that melanocyte migration in the BF might affect B-cell development. Malignant neoplasms of human skin (melanomas) exhibit atypical migration of melanocytes in the lymph nodes^19^,^20^; therefore, the BF acts as an immune organ that provides the material needed to investigate the effects of melanocytes on the development of B-cells in an ideal environment.

RNA-seq allows the rapid exploration of the key genes associated with special phenotypes or important biological processes^21^,^22^,^23^,^24^,^25^. Therefore, RNA-seq was employed to identify a large number of differentially expressed genes, which play important roles in the regulation of cell development in the BF of the three-week-old SF and WL chickens. Key signalling pathways involved in cell growth, proliferation, migration, and fate determination were found to be significantly enriched. These pathways included the cytokine-cytokine receptor interaction, growth hormone-growth hormone receptor interaction, JAK-STAT signalling, WNT signalling, and Fas-FasL receptor interaction pathways, in which the expression of the key genes was found to be significantly reduced in the BF of SF (Fig. S4). Compared to the absence of melanocytes in the BF of WL chickens, the extensive distribution of melanocytes in the BF of SF was closely related to the reduced expression of genes in the above-mentioned pathways, resulting in relatively weaker cell proliferation and development. Wang *et al*.^12^ discovered that the JAK-STAT signalling pathway plays an important role in the regulation of dendritic cell differentiation and function because of the participation of one lncRNA (lncDC)^12^. A recent study reported that the regulation of WNT signalling pathway can maintain hematopoietic stem cells in a quiescent state under homeostatic conditions^26^, indicating that this pathway plays an important role in regulating cell proliferation. In the present study, we detected a reduced expression of the genes associated with the JAK-STAT and WNT signalling pathways in the BF of the three-week-old SF, suggesting that hyperpigmentation affected immature B-cell proliferation and differentiation by regulating these pathways. In addition to the coding genes, the target lncRNA and microRNA genes were found to be enriched in the cell communication, WNT signalling, and JAK-STAT signalling pathways.

In order to better understand the genes expression during the BF development, we further detected the differentially expressed *JAK2, STAT3, IL15*, lncBF2 and lncBF21 in the BF of SF and WL chickens at 0, 2, 6, 10-weeks age. In addition, we detected the expression of these genes in the BF of the black-boned and non-black-boned chickens from the SF and WL hybrid F2 population. As shown in Fig. S5, we observed a relatively lower expression of the *JAK2, STAT3*, and *IL15* genes in the BF of the SFs and F2 black-boned chickens at 2- and 6-weeks age, but the expression of these genes was relatively higher in the BF of SFs at 1-day and 10-weeks age. Higher expression of these genes was greatly related to a large number of melanocytes proliferating in the BF of SF on the hatching day^14^. However, from the age of 2-weeks to 10-weeks, melanocyte migration and melanin synthesis affected B-cell development shown as the decreased expression of these genes in the BF of SF. In addition, an increase in melanin synthesis in melanocytes could generate a large number of reactive oxygen species that adversely affect B-cell proliferation^14^. On the other hand, after the age of 10-weeks, decreased melanocyte proliferation and melanin synthesis resulted in the peaceful coexistence of melanocytes and B-cells, allowing B-cell development in the BF. Additionally, higher lncBF2 and lower lncBF21 expressions were detected in the BF of SFs before 10-weeks age; this might be related to the melanocyte metabolism regulating B-cell development in the BF. However, further studies are needed to confirm the role of noncoding RNAs in the regulation of B-cell development in the BF of SF.

It should be noted that the GO terms of receptor activity, cell communication, and signal transduction as well as the KEGG pathways of cytokine-cytokine receptor interactions, lysosomes, and phagosomes were significantly enriched in the BF of SF. The results indicated the existence of cellular interactions mediated by the cytokines or exosomes in the BF of SF. In addition, a significant change was observed in the expression of the genes related to intercellular regulatory pathways; these genes included the poorly expressed *interleukin* and *interleukin receptor, JAK2*, and *STAT4*, and the highly expressed *TYR* and *TYK2*, suggesting that these genes might be involved in the regulation of melanocytes and immature B-cells. The result of another study from our lab showed that the melanocytes in the BF participated in the inflammatory response during an infectious bursal disease caused by a virus (Fig. S6, data unpublished). Therefore, the melanocytes in the BF also have immune roles, suggesting an interaction between melanocytes and B-cells in the BF of SF. The results of a recent study indicated that the keratinocytes could secrete exosomes, which carry the noncoding RNAs that modulate melanin synthesis in melanocytes after phagocytosis^13^. Moreover, the melanoma cells can secrete exosomes to modulate their microenvironment for metastasis^19^; thus, the melanocytes in the BF might modulate B-cell development depending not only on the cytokines, but also on the noncoding RNAs packaged in the exosomes.

With the exception of developmental pathways, such as the Notch and JAK-STAT signalling pathways, the target lncRNA and microRNA genes, such as the Toll-like and NOD-like receptor genes, were associated with innate immune responses. Moreover, these molecules were also associated with the metabolism of macromolecules, including tryptophan, tyrosine, fatty acid, starch, sucrose, galactose, and retinol. Wu *et al*. (2016) found that aminoglycan, starch, sucrose, and galactose metabolism were all significantly overrepresented, and that the DEGs contribute to body weight heterosis^1^. Thus, the reduced expression of the genes associated with the metabolic pathways in the BF of SF might contribute to a relatively weaker B-cell development and lower BF indices. In addition, in our previous work, we detected low levels of antibodies in SF after vaccination^14^. However, in the present study, the reduced expression of genes was related to the Toll-like and NOD-like receptor pathways, which might result from the proliferation, rather than the differentiation, of B-cells in the BF. Our recent studies also demonstrated that a few immune cells in the cecum of SF were closely related to the reduced expression of sialic acid receptors^27^.

The melanogenesis pathway genes, including *TYR, EDN-3*, and *MC1R*, were significantly highly expressed in the BF of SF. However, the expression of the *Kitlg, Kit*, and *MITF* genes was relatively lower. *TYR, EDN-3*, and *MC1R* are the key genes in melanocytes; however, the genes *Kitlg, Kit*, and *MITF* are not only confined to melanocytes, but also have multiple functions, such as neural crest cell migration^28^ and mast cell development^29^, in other cells. Therefore, the reduced expression of these genes is extremely relevant to the immature B-cell development and mature B-cell migration in the BF of SF.

By analysing the interaction between mRNAs, lncRNAs, and microRNAs, we found that the microRNAs could target lncRNAs and mRNAs and subsequently affect their expression. When microRNAs were used as bridges, the interactions of the targeted lncRNAs and mRNAs were predicted. As shown in Fig. 4, the genes related to cell proliferation, such as *JAK2, STAT3*, and *IL-15*, could be regulated by several lncRNAs and microRNAs. In addition, the relatively lower expressions of the downstream genes associated with cell proliferation, such as *IRF* and *BCL-2*, were detected based on the RNA-seq data. The expression of the *END-3* gene, which is highly associated with melanocyte migration, was relatively higher in the BF of SF, and it was also predicted on the basis of the highly expressed lncRNAs and microRNAs. Therefore, the differentially expressed lncRNAs and microRNAs could regulate gene expression and modulate different biological processes. In the BF of SF, the melanocyte migration and proliferation was accompanied by aberrant B-cell development in a manner similar to the melanocyte migration and function at the site of injury in zebrafish^9^; however, this conclusion requires further study for verification.

## Methods

### Animals

Three female SFs and three female WL chickens (3-weeks old) were obtained from the animal farm of China Agricultural University, Beijing. The protocols for animal use and experimentation were approved by the Beijing Association for Science and Technology (approval ID SYXK [Beijing] 2007–0023), and were in compliance with the Beijing Laboratory Animal Welfare and Ethics guidelines. All animal research work was also approved by the Beijing Administration Committee of Laboratory Animals, and was in accordance with the China Agricultural University (CAU) Institutional Animal Care and Use Committee guidelines (ID: SKLAB-B-2010–003).

### Sample collection

The chickens were sacrificed by severing their jugular veins after anaesthesia, and were bled for 3–5 min before dissection. The BF index was calculated using the following formula: organ index = organ weight/body weight × 100%. The BFs were cut in half along the sagittal plane. One half was fixed in 4% paraformaldehyde, and the other half was stored in liquid nitrogen.

### Immunohistochemistry

The fixed BF samples were trimmed and dehydrated using 30 and 50% sucrose solutions, and were then embedded in the optimum cutting temperature compound (OCT; Leica, Germany) to prepare 5-μm serial sections. As previously reported, the frozen sections were stained with anti-Bu-1 antibodies to label B-cells^14^. The optical densities of the Bu-1^+^ cells and the diameters of bursal nodes were identified in 10 high-power lymph nodes using the Image-Pro Plus 6.0 software, and the means were calculated. Sampling of the coded sections was unbiased, and a single investigator performed the examinations.

### RNA isolation, library preparation, and sequencing

Total RNA was isolated from the BFs of three female SFs and three female WL chickens using the TRIZOL^®^ Reagent (Invitrogen, USA), according to the manufacturer’s instructions. RNA degradation and contamination were visualised on 1% agarose gels. RNA purity was checked using a NanoPhotometer^®^ spectrophotometer (IMPLEN, CA, USA), and concentrations were determined using the Qubit^®^ RNA Assay Kit in Qubit^®^ 2.0 Fluorometer (Life Technologies, CA, USA). RNA integrity was assessed using the RNA Nano 6000 Assay Kit of the Bioanalyzer 2100 system (Agilent Technologies, CA, USA). A total of 3 μg RNA from each sample was used as an input material for RNA sample preparations. The ribosomal RNA was removed using an Epicentre Ribo-zero™ rRNA Removal Kit (Epicentre, WI, USA), and the mRNA and lncRNA sequencing libraries were constructed using an NEBNext^®^ Ultra™ Directional RNA Library Prep Kit for Illumina^®^ (NEB, MA, USA), according to the manufacturer’s recommendations. The microRNA libraries were generated using a NEBNext^®^ Multiplex Small RNA Library Prep Kit for Illumina^®^ (NEB, USA), and index codes were added to attribute the sequences to each sample. The clustering of the index-coded samples was performed on a cBot Cluster Generation System using a TruSeq PE Cluster Kit v3-cBot-HS (Illumina), according to the manufacturer’s instructions. Subsequently, the mRNA and lncRNA libraries were sequenced on an Illumina Hiseq 2000 platform, and 100-bp paired-end reads were generated. The microRNA libraries were sequenced on an Illumina Hiseq 2500/2000 platform, and 50-bp single-end reads were generated.

### Read mapping and identification of differentially expressed mRNAs and lncRNAs

The reference genome and annotation files were downloaded from the *Gallus gallus* genome website (ftp://ftp.ensembl.org/pub/release-74/fasta/gallus_gallus/dna/). The clean data (clean reads) were obtained from the raw reads by removing the reads containing adapters and poly-N regions and low quality reads. The Q20 scores, Q30 scores, and GC content of the clean data were calculated. The reference genome index was constructed using Bowtie v2.0.6, and the clean paired-end reads were aligned to the reference genome using TopHat v2.0.9^30^,^31^. The mapped reads of each sample were assembled by using both Scripture (beta2)^32^ and Cufflinks (v2.1.1)^33^ with a reference-based approach.

The transcripts that were predicted on the basis of the coding potential by either Coding-Non-Coding-Index (CNCI; v2)^34^, Coding Potential Calculator, 0.9-r2 (CPC)^35^, Pfam Scan (v1.3)^36^,^37^, Phylogenetic Codon Substitution Frequency (PhyloCSF; v.20121028)^38^, or all four programs were filtered out, and those without coding potential were designated as the candidate lncRNA set. We used the PhyloFit v1.3 program to compute the phylogenetic models for conserved and non-conserved regions among species. The phastCons program was used in conjunction with the model and HMM transition parameters to compute a set of conservation scores for the coding genes and lncRNAs. The target lncRNA genes were predicted based on the cis and trans orientations^39^. The Cuffdiff (v2.1.1) algorithm was used to calculate the fragments per kilobase of exon per million fragments mapped (FPKMs) of both lncRNAs and coding genes in each sample^40^. Using a model based on the negative binomial distribution^40^, the differentially expressed genes with an adjusted P-value (P_adjust_ < 0.05; Benjamini-Hochberg (BH) multiple test correction) between SF and WL chickens were identified.

### MicroRNA analyses

The clean data were obtained by removing the reads containing poly-N/A/T/G/C regions, 5′ adaptor contamination, low quality reads, or those without 3′ adaptors or insert tags. The Q20 scores, Q30 scores, and GC content of the clean data were also calculated. A specific range, beginning at the clean reads, was chosen for downstream analyses. After being mapped to the reference sequence by Bowtie^41^, the mapped small RNAs were used to search the known microRNAs, and the potential microRNAs and secondary structures were obtained using the modified mirdeep2^42^ and srna-tools-cli tools. The available software tools, miREvo^43^ and mirdeep2^42^, were integrated to predict the novel microRNAs. The microRNA expression levels were estimated through the transcripts per million (TPM) measurements^44^, and the differential expression analyses of the SFs and WL chickens were performed using the DESeq R package (1.8.3). After applying the Benjamini and Hochberg adjustment, a corrected P-value of 0.05 was set as the default threshold for the significantly differential expression. The target microRNA genes were predicted using psRobot-tar in miRanda^45^.

### GO and KEGG enrichment analysis

Gene Ontology (GO) enrichment analyses of the differentially expressed and target genes were implemented using the GOseq R package (Release 2.12)^46^, in which the gene length bias was corrected. The GO terms with a P_adjust_ < 0.05 were considered significantly enriched. We used the KEGG Orthology Based Annotation System (KOBAS v2.0)^47^ software to test the statistical enrichment of the differentially expressed and target genes in Kyoto Encyclopedia of Genes and Genomes (KEGG) pathways in order to understand the high-level functions and utilities of the biological system.

### Interaction of lncRNA, microRNA, and mRNA analyses

The lncRNAs are thought to be pre-microRNAs, and they were examined first because of the high levels of homology. Subsequently, the miRanda software was used to predict the targeted relationships between microRNAs and lncRNAs as well as between microRNAs and mRNAs. As a competing endogenous RNA, a lncRNA can competitively bind microRNA with mRNA; therefore, the lncRNA-microRNA-mRNA pairs were further analysed based on the common microRNA-binding sites^48^.

### Quantitative polymerase chain reaction

For detecting the differentially expressed mRNAs and lncRNAs, total RNA (0.5 μg) from the sequenced BFs was transcribed into cDNA using the Fast Quant RT Kit (with gDNase) (Tiangen Biotech Co., LTD, Beijing, China) according to the manufacturer’s instructions. The expression levels of the five mRNAs and five lncRNAs were quantified with qPCR using the SYBR Green Real-time PCR Master Mix (Vazyme Biotech Co., Ltd, Nanjing, China). The primers for qPCR were designed using the Primer Express 2.0 program (Applied Biosystems, CA, USA), and were subsequently synthesized (Sangon Biotech, Beijing, China). The target mRNAs and lncRNAs are listed in Table 1, and the primer sequences are listed in Table S2. The cycling parameters used for qPCR amplification were as follows: initial heat-denaturation at 95 °C for 4 min; 40 cycles of 95 °C for 30 s, 59 °C for 30 s, and 72 °C for 30 s; and a final extension at 72 °C for 5 min. A melting curve analysis was performed to exclude genomic DNA contamination and to confirm primer specificities. Gene expression was normalised using the 2^−△CT^ method with the glyceraldehyde 3-phosphate dehydrogenase (*GAPDH*) gene as an internal standard. Each biological duplicate was controlled in three technical replicates. For detecting the differentially expressed microRNAs, total RNA (50 ng) from the sequenced BFs was transcribed into cDNA using the TaqMan^®^ MicroRNA Reverse Transcription Kit (Applied Biosystems, USA) according to the manufacturer’s instructions. The expression levels of the five microRNAs were quantified with qPCR using the SYBR Green miRcute Plus miRNA qPCR Detection Kit (Tiangen Biotech Co., LTD., Beijing, China). The primers were designed and synthesized (RIBOBIO CO., LTD, Guangzhou, China). The target microRNAs are listed in Table 1. The cycling parameters used for qPCR amplification were as follows: initial heat-denaturation at 95 °C for 15 min; 45 cycles of 94 °C for 20 s, 60 °C for 34 s. A melting curve analysis was performed to exclude genomic DNA contamination and to confirm primer specificities. Gene expression was normalised using the 2^−△CT^ method with the *U6* gene as an internal standard. Each biological duplicate was controlled in three technical replicates. The high correlation of microRNA expressions from qPCR and RNA-seq was analysed. The Pearson’s correlation between qPCR and RNAseq results was analysed using the software R version 3.2.2.

### Statistical analysis

Data were expressed as means ± standard error (SE). The significance was determined using a one-way analysis of variance (ANOVA) as implemented in the SPSS Software Suite (version 12.0; SPSS Taiwan Corp., Taiwan). Differences were considered statistically significant at P-values < 0.05 and < 0.01.

## Additional Information

**How to cite this article:** Han, D. *et al*. Transcriptome analyses of differential gene expression in the bursa of Fabricius between Silky Fowl and White Leghorn. *Sci. Rep.* **7**, 45959; doi: 10.1038/srep45959 (2017).

**Publisher's note:** Springer Nature remains neutral with regard to jurisdictional claims in published maps and institutional affiliations.

## Supplementary Material

Supplementary Information

## Figures and Tables

**Table 1:** Differentially expressed mRNAs and lncRNAs in BF.

Genes	Gene ID	Gene name	SF read count	WL read count	P_adjust_
mRNA	NM_001030538.1	*JAK2*	1595.46	2882.58	0.001
	NM_001030931.1	*STAT3*	3149.51	5898.76	0.0005
	NM_001001293.1	*GHR*	273.58	676.15	1.22E-06
	NM_204571.1	*IL15*	126.03	454.72	9.71E-08
	NM_204361.1	*KIT*	157.34	315.76	0.0008
lncRNA	TCONS_07444501	*lncBF1*	258.09	20.96	4.93E-28
	TCONS_03572154	*lncBF2*	793.26	80.40	9.25E-21
	TCONS_00007493	*lncBF3*	32.01	0	4.57E-13
	TCONS_05399840	*lncBF4*	61.37	6.72	5.38E-11
	TCONS_03616844	*lncBF22*	0.65	7.90	0.018
microRNAs		gga-miR-204	2610.911	200.5931459	3.28E-19
		*gga-miR-1684b-3p*	70.35429605	0	4.05E-18
		*gga-miR-1551-5p*	36.29889373	2.181201373	8.82E-07
		*novel_34*	97577.95795	31705.58335	7.64E-07
		*gga-miR-146b-3p*	1277.652675	6480.008846	5.50E-09
