# Supplementary material for: Transcriptome analyses of differential gene expression in the bursa of Fabricius between Silky Fowl and White Leghorn
Source: Sci Rep. 2017 Apr 13;7:45959. doi: 10.1038/srep45959 (PMC5390260; doi:10.1038/srep45959)

Transcriptome analyses of differential gene expression in the bursa of Fabricius between Silky Fowl and White Leghorn

Deping Han[1, 2], Yuanyuan Zhang[1], Jianfei Chen[1], Guoying Hua[1], Junying Li[1], Xuegong Deng[3*], Xuemei Deng[1*]

[1]*National Engineering Laboratory for Animal Breeding and Key Laboratory of Animal Genetics, Breeding, and Reproduction of the Ministry of Agriculture, China Agricultural University, Beijing 100193, China;* [2]*College of Veterinary Medicine, China Agricultural University, Beijing, 100193, China;* [3]*College of Science, Northeastern University, Shenyang 110004, China*

*Corresponding authors. dengxuegong@tom.com; deng@cau.edu.cn

Supplementary Figure Legends

Fig. S1. Pearson correlation coefficients for the biological replicates from WL and SF.

Fig. S2. Crucial pathways were clustered from DEGs and lncRNA target genes. GO terms analysis (**A**) and KEGG pathways (**B**) clustered from DEGs. KEGG pathways associated with lncRNA target genes were predicted based on cis (**C**) and trans (**D**) configurations. Red and green indicate lower and higher expression, respectively. The numbers in parentheses indicate the numbers of significantly differentially expressed genes.

Fig. S3. Quantitative PCR validation. The differentially expressed mRNA, lncRNA and microRNA were confirmed by quantitative PCR, and the results from RNA-seq and qPCR were analysed. *P < 0.05, **P < 0.01.

Fig. S4. Genes associated with cell proliferation signalling pathways. Differentially expressed genes in SF were detected and positioned in cell proliferation signalling pathways. Red and green indicate lower and higher expression, respectively, as compared to WL.

Fig. S5. Different genes expressions in BFs of SF and WL aged at different weeks. At 0 weeks old (hatching day), higher expressions of *JAK2*, *STAT3* and lncBF21 were detected in the BF of SF. At weeks 2 and 6, lower expressions of *JAK2*, *STAT3* and lncBF21 were found in the BF of SF and black-boned chicken from the WL and SF hybrid F2 population (6 weeks old). But higher expression of was detected in the BF of SF at 2 weeks old. At 10 weeks old, there were higher expressions of *JAK2*, *STAT3* and lncBF2 and lower expression of lncBF21 in the BF of SF. *P < 0.05, **P < 0.01. B,

Black-boned chicken. Non B, non-Black-boned chicken.

Fig. S6. Changes of melanocytes in BF of SF after infectious bursal disease virus infection. Control, no infection. 5DPI, 5 days post infection. 7DPI, 7 days post infection. Melanocyte (red arrow), heterophilic granulocyte (blue arrow) and apoptotic cell (green arrow) were observed in the BF.

Table S1. Primers used to detect mRNA and lncRNA expression.

Genes	Gene name	Primers	Length
mRNA	*Jak2*	F: 5′-ACGACTGCCAAGACCAGATG-3′<br>R: 5′-TTCCTCACCCACCCATGTTG-3′	128
	STAT3	F: 5′-CGCACCCTTGACTCACTCAT-3′<br>R: 5′-TTGGTGAGGAAGCACACTCC-3′	134
	GHR	F: 5′-ACACAGATACCCAACAGCCG-3′<br>R: 5′-ACCTTGGATTTCTGCCCTGG-3′	145
	IL15	F: 5′-GGCCGGAGAGTCAGAAAACA-3′<br>R: 5′-TGTCTTTGGTACATAAGCACATAGG-3′	176
	KIT	F: 5′-AAGTGGGAAGCCAAGGGATG-3′<br>R: 5′-CGATTCTGACTGCGGTGGAT-3′	124
lncRNA	*lncBF1*	F: 5′-AAAAGCCGGGGTCAAAAGGA-3′<br>R: 5′TTTGAGAACCACAGGGTCGG-3′	209
	lncBF2	F: 5′-AGACCTGCAGAGCTAAGGGA-3′<br>R: 5′ACCAGGAAAACACGATGCCT-3′	185
	lncBF3	F: 5′-ACGTGATGTCAAACAGCGTTC-3′<br>R: 5′GCCTGTAGGTGAGTTCTGCT-3′	201
	lncBF4	F: 5′-GGGCAAGTTATGCTGTGTGC-3′<br>R: 5′AGATAGCACCCTGCTTCGTG-3′	274
	lncBF22	F: 5′-ACTGAGCATGGCAAGAGTCC-3′<br>R: 5′CCAAAGAGGAGCTCCCACAG-3′	278

lncBF: long noncoding RNA in the bursa of Fabricius.

Figure-S1

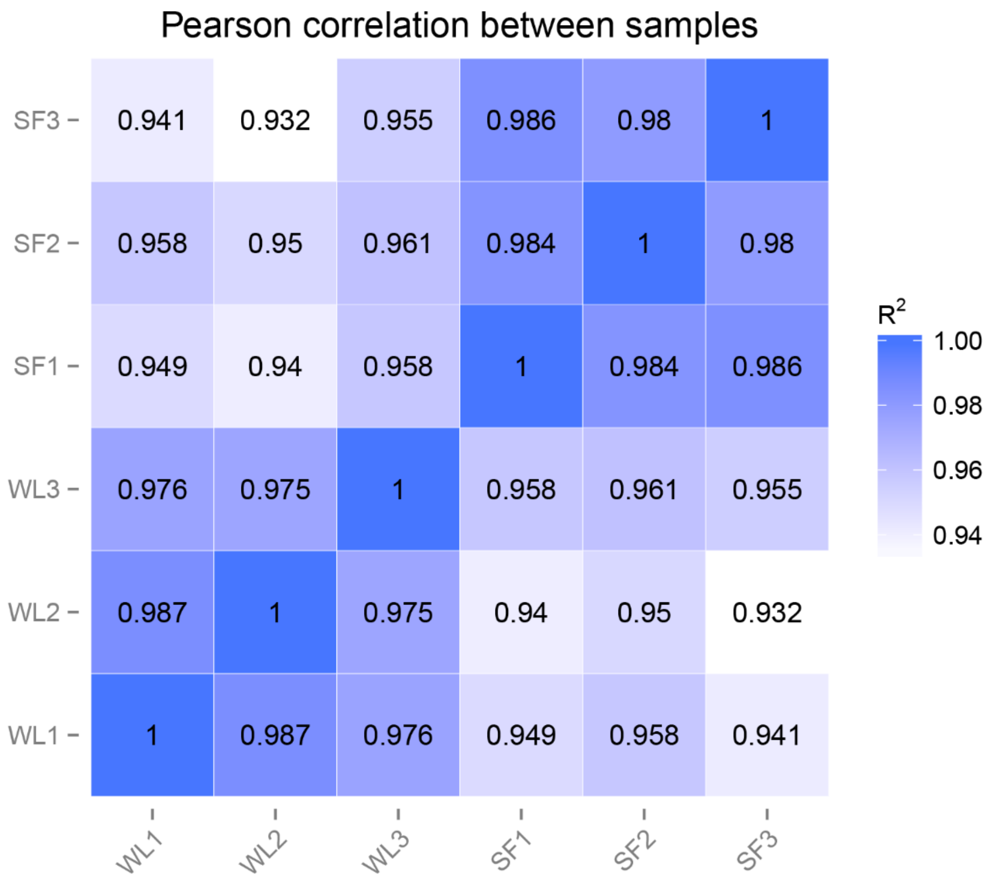
Pearson correlation between samples
SF3
SF2
SF1
WL3
WL2
WL1
0.941
0.932
0.955
0.986
0.98
1
0.958
0.95
0.961
0.984
1
0.98
0.949
0.94
0.958
1
0.984
0.986
0.976
0.975
1
0.958
0.961
0.955
0.987
1
0.975
0.94
0.95
0.932
1
0.987
0.976
0.949
0.958
0.941
WL1
WL2
WL3
SF1
SF2
SF3
R2
1.00
0.98
0.96
0.94

Figure-S2

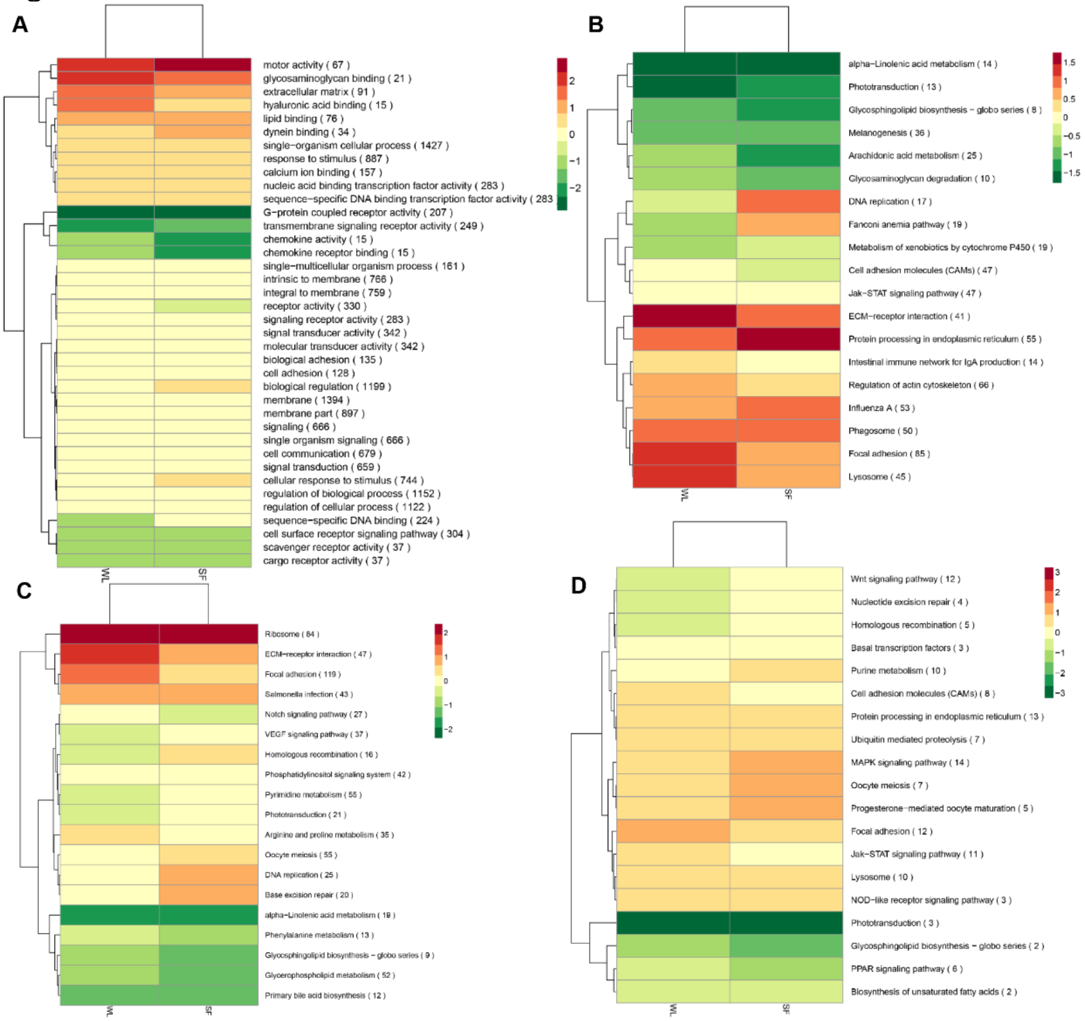
A
motor activity (67)
glycosaminoglycan binding (21)
extracellular matrix (91)
hyaluronic acid binding (15)
lipid binding (76)
dynein binding (34)
single−organism cellular process (1427)
response to stimulus (887)
calcium ion binding (157)
nucleic acid binding transcription factor activity (283)
sequence−specific DNA binding transcription factor activity (283
G−protein coupled receptor activity (207)
transmembrane signaling receptor activity (249)
chemokine activity (15)
chemokine receptor binding (15)
single−multicellular organism process (161)
intrinsic to membrane (766)
integral to membrane (759)
receptor activity (330)
signaling receptor activity (283)
signal transducer activity (342)
molecular transducer activity (342)
biological adhesion (135)
cell adhesion (128)
biological regulation (1199)
membrane (1394)
membrane part (897)
signaling (666)
single organism signaling (666)
cell communication (679)
signal transduction (659)
cellular response to stimulus (744)
regulation of biological process (1152)
regulation of cellular process (1122)
sequence−specific DNA binding (224)
cell surface receptor signaling pathway (304)
scavenger receptor activity (37)
cargo receptor activity (37)
2
1
0
−1
−2
WL
SF
B
alpha−Linolenic acid metabolism (14)
Phototransduction (13)
Glycosphingolipid biosynthesis − globo series (8)
Melanogenesis (36)
Arachidonic acid metabolism (25)
Glycosaminoglycan degradation (10)
DNA replication (17)
Fanconi anemia pathway (19)
Metabolism of xenobiotics by cytochrome P450 (19)
Cell adhesion molecules (CAMs) (47)
Jak−STAT signaling pathway (47)
ECM−receptor interaction (41)
Protein processing in endoplasmic reticulum (55)
Intestinal immune network for IgA production (14)
Regulation of actin cytoskeleton (66)
Influenza A (53)
Phagosome (50)
Focal adhesion (85)
Lysosome (45)
1.5
1
0.5
0
−0.5
−1
−1.5
WL
SF
C
Ribosome (84)
ECM−receptor interaction (47)
Focal adhesion (119)
Salmonella infection (43)
Notch signaling pathway (27)
VEGF signaling pathway (37)
Homologous recombination (16)
Phosphatidylinositol signaling system (42)
Pyrimidine metabolism (55)
Phototransduction (21)
Arginine and proline metabolism (35)
Oocyte meiosis (55)
DNA replication (25)
Base excision repair (20)
alpha−Linolenic acid metabolism (19)
Phenylalanine metabolism (13)
Glycosphingolipid biosynthesis − globo series (9)
Glycerophospholipid metabolism (52)
Primary bile acid biosynthesis (12)
2
1
0
−1
−2
WL
SF
D
Wnt signaling pathway (12)
Nucleotide excision repair (4)
Homologous recombination (5)
Basal transcription factors (3)
Purine metabolism (10)
Cell adhesion molecules (CAMs) (8)
Protein processing in endoplasmic reticulum (13)
Ubiquitin mediated proteolysis (7)
MAPK signaling pathway (14)
Oocyte meiosis (7)
Progesterone−mediated oocyte maturation (5)
Focal adhesion (12)
Jak−STAT signaling pathway (11)
Lysosome (10)
NOD−like receptor signaling pathway (3)
Phototransduction (3)
Glycosphingolipid biosynthesis − globo series (2)
PPAR signaling pathway (6)
Biosynthesis of unsaturated fatty acids (2)
3
2
1
0
−1
−2
−3
WL
SF

Figure-S3

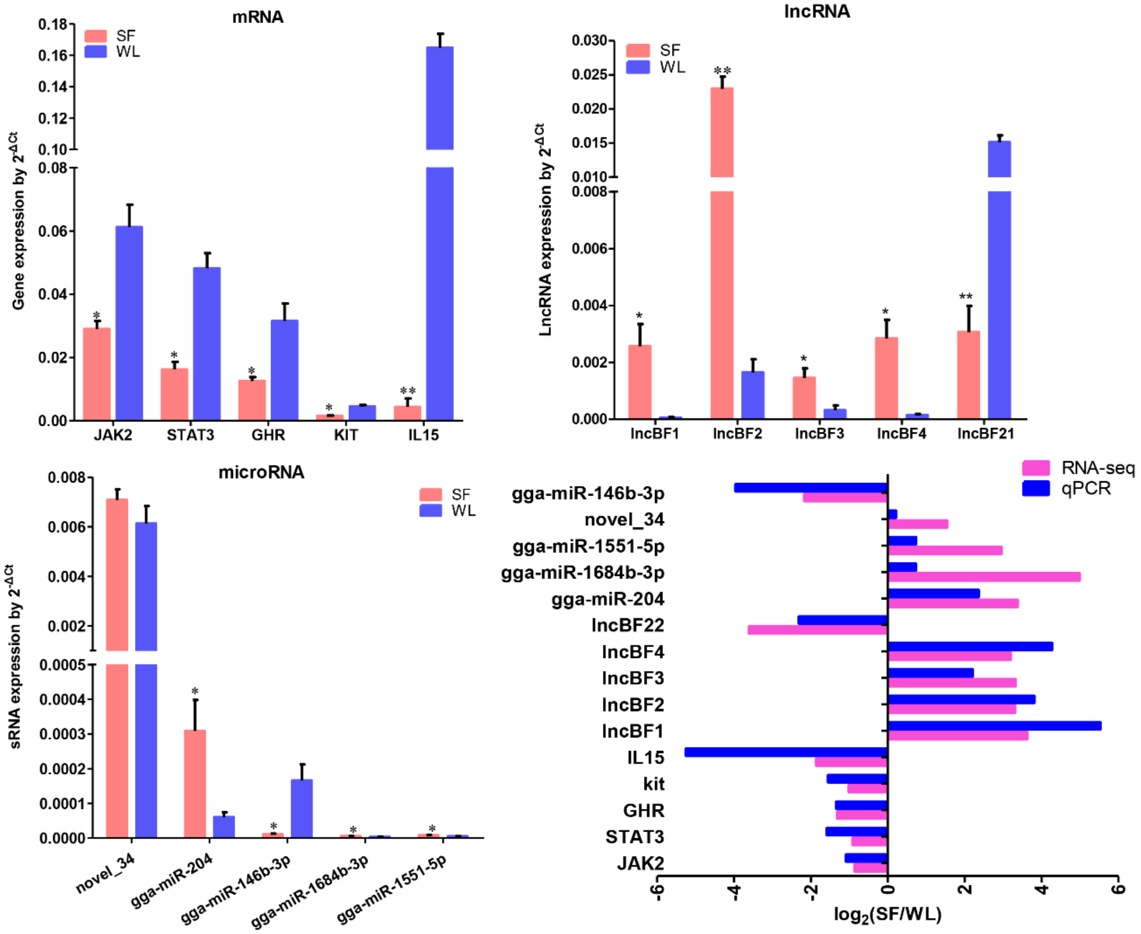

mRNA
SF
WL
Gene expression by $2^{-\Delta Ct}$
0.18
0.16
0.14
0.12
0.10
0.08
0.06
0.04
0.02
0.00
JAK2
STAT3
GHR
KIT
IL15
lncRNA
LncRNA expression by $2^{-\Delta Ct}$
0.030
0.025
0.020
0.015
0.010
0.008
0.006
0.004
0.002
0.000
lncBF1
lncBF2
lncBF3
lncBF4
lncBF21
microRNA
sRNA expression by $2^{-\Delta Ct}$
0.008
0.006
0.004
0.002
0.0005
0.0004
0.0003
0.0002
0.0001
0.0000
novel_34
gga-miR-204
gga-miR-146b-3p
gga-miR-1684b-3p
gga-miR-1551-5p
RNA-seq
qPCR
gga-miR-146b-3p
novel_34
gga-miR-1551-5p
gga-miR-1684b-3p
gga-miR-204
lncBF22
lncBF4
lncBF3
lncBF2
lncBF1
IL15
kit
GHR
STAT3
JAK2
-6
-4
-2
0
2
4
6
$\log_2(SF/WL)$

Figure-S4

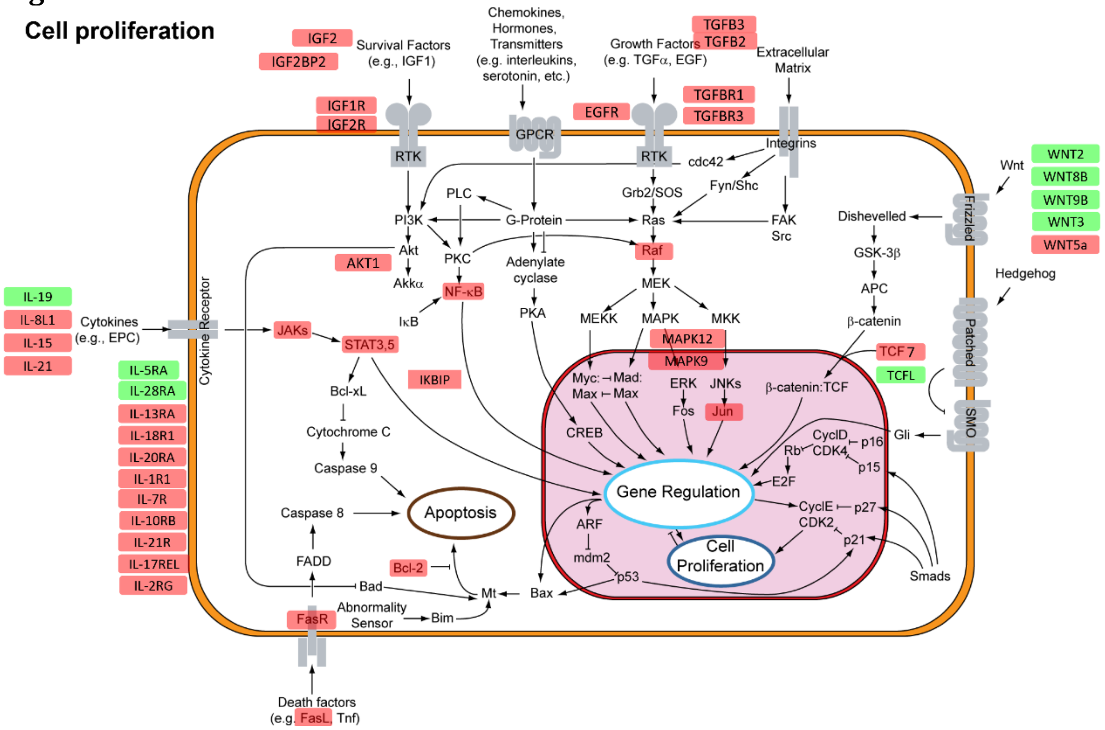
Cell proliferation
IGF2
IGF2BP2
Survival Factors (e.g., IGF1)
Chemokines, Hormones, Transmitters (e.g. interleukins, serotonin, etc.)
Growth Factors (e.g. TGFα, EGF)
TGFB3
TGFB2
Extracellular Matrix
IGF1R
IGF2R
EGFR
TGFBR1
TGFBR3
RTK
GPCR
RTK
Integrins
cdc42
Fyn/Shc
Grb2/SOS
PLC
PI3K
G-Protein
Ras
FAK
Src
Wnt
WNT2
WNT8B
WNT9B
WNT3
WNT5a
Frizzled
Dishevelled
GSK-3β
APC
β-catenin
Hedgehog
Patched
SMO
Akt
AKT1
Akkα
PKC
Adenylate cyclase
Raf
MEK
NF-κB
IκB
PKA
MEKK
MAPK
MKK
MAPK12
MAPK9
IL-19
IL-8L1
IL-15
IL-21
Cytokines (e.g., EPC)
Cytokine Receptor
IL-5RA
IL-28RA
IL-13RA
IL-18R1
IL-20RA
IL-1R1
IL-7R
IL-10RB
IL-21R
IL-17REL
IL-2RG
JAKs
STAT3,5
IKBIP
Bcl-xL
Cytochrome C
Caspase 9
Caspase 8
Apoptosis
FADD
Bcl-2
Bad
Mt
Bax
Bim
FasR
Abnormality Sensor
Death factors (e.g. FasL, Tnf)
Myc: Max
Mad: Max
ERK
Fos
JNKs
Jun
β-catenin:TCF
TCF 7
TCFL
CREB
Gli
CyclD CDK4
p16
Rb
p15
E2F
Gene Regulation
CyclE CDK2
p27
ARF
mdm2
p53
p21
Cell Proliferation
Smads

Figure-S5

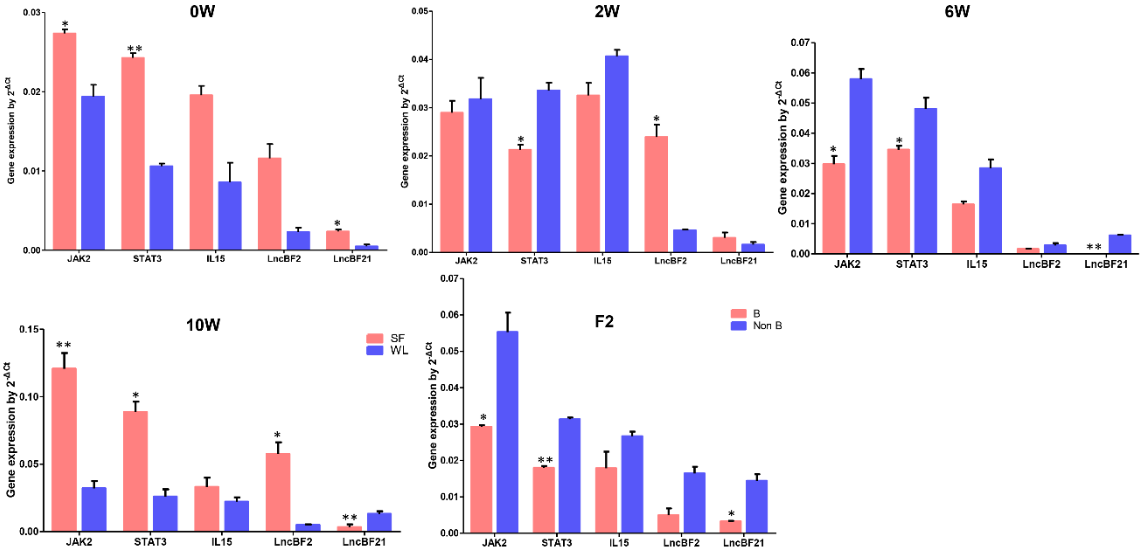
0W
2W
6W
10W
F2
Gene expression by $2^{-\Delta Ct}$
JAK2
STAT3
IL15
LncBF2
LncBF21
SF
WL
B
Non B

Figure-S6

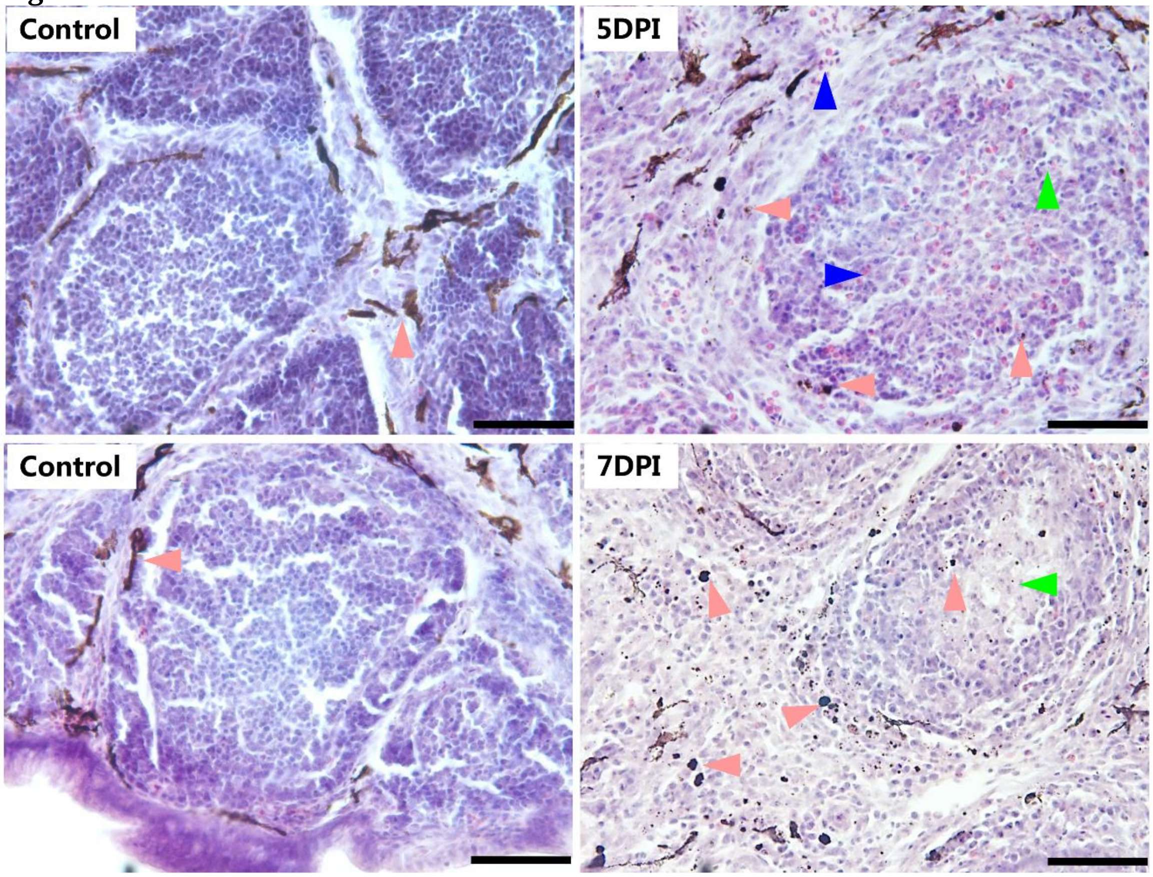

Supplement: Supplementary Information [file srep45959-s1.pdf]
